# Association of Helicobacter Pylori Infection and Colon Cancer

**DOI:** 10.4021/jocmr880w

**Published:** 2012-05-15

**Authors:** Alexandros Strofilas, Emmanuel E Lagoudianakis, Charalambos Seretis, Apostolos Pappas, Nikolaos Koronakis, Dimitrios Keramidaris, Ilias Koukoutsis, Ioannis Chrysikos, Ioannis Manouras, Andreas Manouras

**Affiliations:** a Second Department of Surgery, 401 Army General Hospital, Athens, Greece; b First Department of Propaedeutic Surgery, Hippocrateion Hospital, Athens Medical School, Athens, Greece; c Second Department of Surgery, 417 NIMTS-Nosileutiko Idrima Metohikou Tameiou Stratou (Military Veterans' Fund Hospital), Athens, Greece

**Keywords:** Gastrin, Colorectal cancer, Helicobacter pylori, Cag+

## Abstract

**Background:**

Gastrin has been shown to exert carcinogenic effect to the epithelium of the colon. This study examines whether hypergastrinemia and H. pylori infection -especially infection by the CagA+ strain- are statistically associated with colorectal cancer and examine possible correlations with the colorectal cancer stage and lymph node metastasis.

**Methods:**

In this prospective case-control study, fasting serum samples from 93 consecutive patients with colorectal cancer treated in a university surgical clinic were preoperatively collected and serum levels of gastrin were measured. A group of 20 age matched hernia patients were used as controls. The pathology report of the specimens was documented and statistical analysis of the data where performed with the spss 17 statistical suite.

**Results:**

H. pylori IgG antibodies was reported in 66/93 (71%) in the colorectal cancer group and 13/20 patients in the control group (65%), the difference having non-statistical significance (P = n.s). The prevalence of cagA protein expression in the anti- H. pylori IgG+ patients were higher in the colorectal cancer group (56% positivity), when compared to the control group (38,4% positivity) but the difference was not of statistical significance (P = n.s). The mean levels of serum gastrin levels in the two groups did not significantly differ (Ca group 51.1 ± 36.6 pg/mL vs Control 49.8 ± 17.6 P = n.s.). Patients with lymph node metastasis had higher serum gastrin levels than patients without metastasis and this difference was statistically significant. (53.6 vs 41.06 pg/mL P = 0.025).

**Conclusions:**

Although the serum gastrin levels were not statistically different between the TNM stages of our patient cohort, our data found that serum gastrin levels were significantly higher in patients with lymph node metastasis. Whether gastrin is implicated in the ability of cancer cells to metastasize to the lymph nodes merits further research.

## Introduction

Helicobacter pylori infection and hypergastrinemia are considered to be related to the development of colorectal cancer. However, the results of previously conducted studies showed controversial findings, not being able to either reject or clearly support a direct correlation between H. pylori infection and/or elevated gastrin levels with colorectal carcinogenesis.

H. pylori is recognized as a class I human carcinogen, according to the International Agency for Research on Cancer [1]. Although, H. pylori infection has been causative associated with gastric carcinoma and lymphoma, its role in colorectal cancer development remains unclarified [2]. A possible pathogenetic mechanism involves the persistent H. pylori colonization and inflammation of the gastric mucosa, particularly when the H. pylori strains express the cytoxin-associated gene (CagA) which often results in the development of chronic atrophic gastritis and subsequently hypergastrinemia, through a reverse-feedback mechanism; hypergastrinemia is considered to be a possible risk factor for the development of colorectal cancer [3-5].

The aim of our case-control study is to examine whether hypergastrinemia and H. pylori infection -especially infection by the CagA+ strain- are statistically associated with colorectal cancer and examine possible correlations with the colorectal cancer stage and lymph node metastasis.

## Methods

The study population of this prospective case-control study included colorectal cancer patients admitted for surgical treatment in a university hospital's surgical department and a demographically matched patient population who were scheduled for hernia repair as a control group.

Fasting serum samples were obtained from all patients, in order to assay anti-H. pylori immunoglobulin G, CagA protein expression and serum gastrin levels. An enzyme-linked immunosorbent assay (ELISA) IgG serologic test for H pylori diagnosis (HEL-P test, Park Co, Athens, Greece) was used for the detection of H. pylori positive patients in accordance with the manufacturer's guidelines. A positive, result was assigned when the concentration of IgG antibodies against H pylori was greater than 25 U/mL. The positive serums were further examined by western-blot IgG assay for the cytotoxin-associated gene A (CagA) protein. Gastrin in serum was assayed by a competitive radioimmunoassay using a rabbit antiserum raised against a gastrin 17 albumin conjugate (Gastrin RIA, DIAsource ImmunoAssays S.A, Belgium). The antiserum used in this assay cross reacts with gastrin-34 and the sulphated forms of gastrin-17 and gastrin-34. Normal were considered when values < 100 pg/mL.

In all cancer patients colorectal malignancy was identified by colonoscopy and histopathological examination of the biopsy specimens which were obtained during endoscopy.

Exclusion criteria were set in order to achieve the minimal effect of external factors regarding the fluctuation of the parameters measured. Patients who were receiving gastric antisecretory medication (proton pump inhibitors, H_2_ blockers) and non-steroidal anti-inflammatory drugs were excluded from the study. Moreover, exclusion criteria met the positive history of gastroduodenal surgical operations, Zollinger-Ellison syndrome and alcohol addiction, so as to minimize the impact of the factors above as false positive causes of hypergastrinemia.

## Results

The colorectal cancer patient group comprised from 93 patients with mean age of 69.1 ± 10.1 years, while the mean age of the control group was 64.2 ± 14.8 years, the difference being non-significant.

Positivity of anti- H. pylori IgG antibodies was reported in 66/93 (71%) in the colorectal cancer group, while in the control group presence of the antibodies was detected in 13/20 patients (65%), the difference having non-statistical significance (P = n.s.).

The prevalence of cagA protein expression in the anti- H. pylori IgG+ patients were higher in the colorectal cancer group (56% positivity), when compared to the control group (38.4% positivity). However, the difference was not of statistical significance (P = n.s).

The mean levels of serum gastrin levels in the two groups did not significantly differ (Ca group 51.1 ± 36.6 pg/mL vs Control 49.8 ± 17.6, P = n.s.). Furthermore, the mean gastrin levels were not significantly higher in patients with colorectal cancer and H. pylori infection (mean value: 51 pg/mL), compared with the negative for infection colorectal cancer patients (mean value: 51.6 pg/mL) and the control group (P = n.s.).

**Figure 1:**
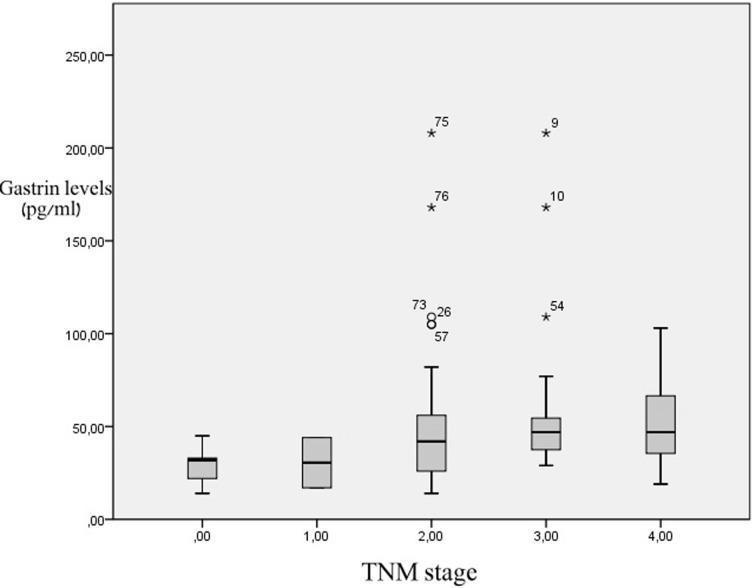
Serum gastrin levels in relation to colon cancer stage. There are no statistical differences between groups.

The analysis showed that there was no statistically significant difference of the serum gastrin levels in the patients with colorectal malignancy in terms of the location of the primary lesion. Similarly, no statistical significance was found when the TNM stage of colorectal cancer was correlated to serum gastrin levels (Fig. 1). On the contrary, patients with infiltrated lymph nodes had higher gastrin levels than the patients without metastases, with the results reaching statistical significance (53.6 pg/mL vs 41.06 pg/mL, P = 0.025).

## Discussion

The role of H. pylori infection and hypergastrinemia in the development of colorectal carcinogenesis has been a matter of scientific debate, with controversial findings up to present. Gastrin is a 17-aminoacid polypeptide, produced by the G-cells of the gastric antrum, duodenum and pancreas. The hormone is involved in the secretion of gastric acid from the parietal cells of the stomach, as well as in the regulation of pancreatic enzyme activity and also appears to play an important role in the modulation of gastric emptying [6]. As it comes to colorectal cancer, hypergastrinemia has been reported to be associated with increased risk for colorectal malignancy, as the hormone is considered to serve as a growth factor of normal colonic epithelium [7-9]. In addition, it appears that patients with elevated gastrin levels due to Zollinger-Ellison syndrome have higher markers of colonic proliferation [10]. On the other hand, in a study by Orbuch et al (1996), performed similarly in patients with Zollinger-Ellison syndrome, the results showed no significant correlation between hypergastrinemia and increased risk of colorectal cancer [11]. Moreover, Bombski et al (2003) have demonstrated a decline in preoperatively elevated serum gastrin levels after curative surgical operations in colorectal cancer patients [12].

The exact mechanism through which gastrin acts in the progress of colorectal carcinogenesis remains a matter of dispute. Many authors have suggested that gastrin is produced and secreted by the malignant intestinal cells and demonstrate its growth stimulation effects via autocrine or paracrine pathways [13, 14]. Focusing in a more molecular basis, recent studies have proven that gastrin is related to upregulation of expression of inflammatory mediators, such as COX-2 and IL-8, whose inhibition can contribute in the prevention of colorectal cancer development [15]. In addition, there seems to be a genetic bridge between hypergastrinemia and colorectal cancer, as gastrin expression is induced by the k-ras oncogene [16]. Lastly, there is a mounting scientific debate concerning the role of gastrin precursors, such as gastrin-gly, which seem to upregulate proangiogenic factors, like VEGF in colon cancer cells [17]. Based upon these recent data, it is not surprising that gastrin precursors and not gastrin itself may be involved in colorectal cancer, bridging the gap regarding the presence of hypergastrinemia in colorectal cancer patients. Nevertheless, the exact significance of gastrin or its precursors in colorectal cancer is still a field of scientific investigation and safe conclusions cannot be reached yet.

Our results did not demonstrate a statistically significant difference of serum gastrin levels in the colorectal cancer group compared to controls. However, we did show that there was a statistically significant increase of serum gastrin in patients with lymph node metastases. The measurement of gastrin as a predictor of colorectal metastatic lesions in the liver has been proposed by Kameyama et al (1993) [18]. Moreover, the same research group has reported positive effect of administration of gastrin antagonists after liver resection due to colorectal metastatic disease [19].

Another matter of conflict remains the possible correlation of gastrin levels and colorectal cancer stage. Our study results were not supportive of a positive correlation between gastrin serum levels and the TNM stage of the tumor, as proposed in some published studies [20-22]. Similar conclusions with our results have reached the study groups of Fontanesi et al. and Vanderstraeten et al [23, 24]. However, the clinical significance of these findings cannot at present be safely interpreted, since the underlying pathophysiological pathways remain terra incognita.

Helicobacter pylori infection is one of the most common causes of hypergastrinemia and chronic atrophic gastritis and also is firmly associated with gastroduodenal ulceration, gastric carcinoma and lymphoma, with about 50% of the worldwide population being carriers of the pathogen [23]. In addition, H. pylori strains that express the cytoxin-associated gene (CagA+) are considered to further induce the inflammatory stress in the colonized gastric mucosa, compared to the CagA- H. pylori strains, leading more frequently to the development of chronic atrophic gastritis, condition directly related to elevation of gastrin levels, through reverse feedback regulation [24-26]. Moreover, if chronic atrophic gastritis is established, the following hypochlorhydria may result in intestinal overgrowth of acid-sensitive microflora, which has also been implicated in colorectal cancer progression [27], fact that also proposes a potential link between colorectal cancer and elevated gastrin levels. The main pathogenic mechanisms which are considered to be involved include the occurring hypergastrinemia in cases of atrophic gastritis, as described above, the increase of the intraluminal ammonia products, which can trigger intracellular tumorigenic mechanisms, and finally the promotion of systemic inflammation, through overexpression of proinflammatory cytokines (IL-1, IL-8, TNF-α, etc) and growth factors (EGF, TGF-α, etc) [28]. It is accepted that H. pylori strains that express the cytoxin-associated gene (CagA+) are associated to even greater increase of local and systemic inflammation [29], rising speculations regarding a possible correlation of increased seroprevalence of CagA+ strains in colorectal cancer patients, compared to CagA- strains. In our study, the prevalence of H. pylori antibodies did not statistically differ between the two groups, in accordance to numerous previous case-control studies [30-33]. However, there are sufficient published data, supportive of increased H. pylori seroprevalence in colorectal cancer patients, highlighting the impact of the pathophysiological mechanisms described above as favorable for the development of colorectal neoplasia [34-36]. Although recent meta-analyses indicate that H. pylori infection slightly increases the risk for the development of colorectal cancer (OR increased from 1.36 to 1.58) [2, 28], more studies need to be performed in order to either confirm or reject the association between H. pylori infection and colorectal cancer.

Our results did not show a statistically significant increased in the seroprevalence of CagA+ strains in the colorectal cancer group. This finding is contradictory with the results of the research groups of Shmuely et al (2001) and Hartwich et al, who reported that CagA+ seropositivity is associated with increased risk for both gastric and colonic cancer [35, 37]. It would be challenging to speculate that the enhanced inflammation status, both systemic and gastric, caused by the CagA+ strains could trigger the systematic cytokines cascade, predisposing to the formation of premalignant lesions. Focusing in the focal gastric inflammation, the CagA+ strains, compared to CagA-, are associated with increased hazard of chronic atrophic gastritis and consequently hypergastrinemia, which appears to be related to colorectal carcinogenesis, directly or indirectly. At last, it can be concluded that CagA+ strains may exert the metabolism of ammonia products in the large intestine, which can act as endogenous carcinogens, although further studies are required to confirm our hypotheses.

The significance of gastrin and H. pylori infection in colorectal cancer progress still appears like a never-ending conquest. Our study did not reveal statistically significant differences in gastrin levels among colorectal cancer patients and controls. Additionally, no correlation occurred between gastrin levels and the site of colorectal cancer or the stage of the disease. Nevertheless, it appeared that in colorectal cancer patients with infiltrated lymph nodes, the levels of gastrin were statistically significantly higher compared to the patients with no lymph node infiltrations. It would be interesting to investigate in future studies the potential role of gastrin in the mechanisms of metastasis of the tumor cells. Regarding the seroprevalence of H. pylori infection in colorectal cancer patients, no statistically significance occurred between the two groups of our study. Moreover, therewere no statistical significant difference in the prevalence of the CagA serotype in the colorectal cancer group, when compared to the control group. The specific role of helicobacter pylori CagA+ strains should be examined further in the effort to elucidate the impact of h. pylori infection in the colorectal cancer progress and also to examine the potential of being a target towards the inhibition of colorectal cancerogenesis.

## References

[1] WHO (1994). IARC monographs on the evaluation of carcinogenic risks to humans: schistosomes, liver flukes and Helicobacter pylori. Vol 6, Lyon, France: IARC.

[2] Zumkeller N, Brenner H, Zwahlen M, Rothenbacher D (2006). Helicobacter pylori infection and colorectal cancer risk: a meta-analysis. Helicobacter.

[3] Hakanson R, Sundler F (1991). Trophic effects of gastrin. Scand J Gastroenterol Suppl.

[4] Ryberg B, Axelson J, Hakanson R, Sundler F, Mattsson H (1990). Trophic effects of continuous infusion of [Leu15]-gastrin-17 in the rat. Gastroenterology.

[5] Renga M, Brandi G, Paganelli GM, Calabrese C, Papa S, Tosti A, Tomassetti P (1997). Rectal cell proliferation and colon cancer risk in patients with hypergastrinaemia. Gut.

[6] D'Onghia V, Leoncini R, Carli R, Santoro A, Giglioni S, Sorbellini F, Marzocca G (2007). Circulating gastrin and ghrelin levels in patients with colorectal cancer: correlation with tumour stage, Helicobacter pylori infection and BMI. Biomed Pharmacother.

[7] Creutzfeldt W, Lamberts R (1991). Is hypergastrinaemia dangerous to man?. Scand J Gastroenterol Suppl.

[8] Sirinek KR, Levine BA, Moyer MP (1985). Pentagastrin stimulates in vitro growth of normal and malignant human colon epithelial cells. Am J Surg.

[9] Chu M, Rehfeld JF, Borch K (1992). Effects of gastric fundectomy and antrectomy on the colonic mucosa in the hamster. Digestion.

[10] Sobhani I, Lehy T, Laurent-Puig P, Cadiot G, Ruszniewski P, Mignon M (1993). Chronic endogenous hypergastrinemia in humans: evidence for a mitogenic effect on the colonic mucosa. Gastroenterology.

[11] Orbuch M, Venzon DJ, Lubensky IA, Weber HC, Gibril F, Jensen RT (1996). Prolonged hypergastrinemia does not increase the frequency of colonic neoplasia in patients with Zollinger-Ellison syndrome. Dig Dis Sci.

[12] Bombski G, Gasiorowska A, Orszulak-Michalak D, Neneman B, Kotynia J, Strzelczyk J, Janiak A (2003). Elevated plasma gastrin, CEA, and CA 19-9 levels decrease after colorectal cancer resection. Int J Colorectal Dis.

[13] Hollande F, Imdahl A, Mantamadiotis T, Ciccotosto GD, Shulkes A, Baldwin GS (1997). Glycine-extended gastrin acts as an autocrine growth factor in a nontransformed colon cell line. Gastroenterology.

[14] Hoosein NM, Kiener PA, Curry RC, Brattain MG (1990). Evidence for autocrine growth stimulation of cultured colon tumor cells by a gastrin/cholecystokinin-like peptide. Exp Cell Res.

[15] Chao C, Hellmich MR (2010). Gastrin, inflammation, and carcinogenesis. Curr Opin Endocrinol Diabetes Obes.

[16] Aly A, Shulkes A, Baldwin GS (2004). Gastrins, cholecystokinins and gastrointestinal cancer. Biochim Biophys Acta.

[17] Bertrand C, Kowalski-Chauvel A, Do C, Resa C, Najib S, Daulhac L, Wang TC (2010). A gastrin precursor, gastrin-gly, upregulates VEGF expression in colonic epithelial cells through an HIF-1-independent mechanism. Int J Cancer.

[18] Kameyama M, Fukuda I, Imaoka S, Nakamori S, Iwanaga T (1993). Level of serum gastrin as a predictor of liver metastasis from colorectal cancer. Dis Colon Rectum.

[19] Kameyama M, Nakamori S, Imaoka S, Yasuda T, Nakano H, Ohigashi H, Hiratsuka M (1994). [Adjuvant chemo-endocrine chemotherapy with gastrin antagonist after resection of liver metastasis in colorectal cancer]. Gan To Kagaku Ryoho.

[20] Smith JP, Wood JG, Solomon TE (1989). Elevated gastrin levels in patients with colon cancer or adenomatous polyps. Dig Dis Sci.

[21] Upp JR, Jr, Singh P, Townsend CM, Jr, Thompson JC (1989). Clinical significance of gastrin receptors in human colon cancers. Cancer Res.

[22] Wong K, Beardshall K, Waters CM, Calam J, Poston GJ (1991). Postprandial hypergastrinaemia in patients with colorectal cancer. Gut.

[23] Fontanesi BV, Boris B, Delcio M, Manoukian FN (1997). Gastrin levels in patients with colorectal cancer. Hepatogastroenterology.

[24] Vanderstraeten EF, De Vos MM, Versieck JM, Elewaut AP (1995). Serum gastrin levels and colorectal neoplasia. Dis Colon Rectum.

[25] Limburg PJ, Stolzenberg-Solomon RZ, Colbert LH, Perez-Perez GI, Blaser MJ, Taylor PR, Virtamo J (2002). Helicobacter pylori seropositivity and colorectal cancer risk: a prospective study of male smokers. Cancer Epidemiol Biomarkers Prev.

[26] Kim JH, Park HJ, Cho JS, Lee KS, Lee SI, Park IS, Kim CK (1999). Relationship of CagA to serum gastrin concentrations and antral G, D cell densities in Helicobacter pylori infection. Yonsei Med J.

[27] Peek RM, Jr, Miller GG, Tham KT, Perez-Perez GI, Zhao X, Atherton JC, Blaser MJ (1995). Heightened inflammatory response and cytokine expression in vivo to cagA+ Helicobacter pylori strains. Lab Invest.

[28] Zhao YS, Wang F, Chang D, Han B, You DY (2008). Meta-analysis of different test indicators: Helicobacter pylori infection and the risk of colorectal cancer. Int J Colorectal Dis.

[29] Machida-Montani A, Sasazuki S, Inoue M, Natsukawa S, Shaura K, Koizumi Y, Kasuga Y (2007). Atrophic gastritis, Helicobacter pylori, and colorectal cancer risk: a case-control study. Helicobacter.

[30] Siddheshwar RK, Muhammad KB, Gray JC, Kelly SB (2001). Seroprevalence of Helicobacter pylori in patients with colorectal polyps and colorectal carcinoma. Am J Gastroenterol.

[31] Aydin A, Karasu Z, Zeytinoglu A, Kumanlioglu K, Ozacar T (1999). Colorectal adenomateous polyps and Helicobacter pylori infection. Am J Gastroenterol.

[32] Moss SF, Neugut AI, Garbowski GC, Wang S, Treat MR, Forde KA (1995). Helicobacter pylori seroprevalence and colorectal neoplasia: evidence against an association. J Natl Cancer Inst.

[33] Thorburn CM, Friedman GD, Dickinson CJ, Vogelman JH, Orentreich N, Parsonnet J (1998). Gastrin and colorectal cancer: a prospective study. Gastroenterology.

[34] Meucci G, Tatarella M, Vecchi M, Ranzi ML, Biguzzi E, Beccari G, Clerici E (1997). High prevalence of Helicobacter pylori infection in patients with colonic adenomas and carcinomas. J Clin Gastroenterol.

[35] Hartwich A, Konturek SJ, Pierzchalski P, Zuchowicz M, Labza H, Konturek PC, Karczewska E (2001). Helicobacter pylori infection, gastrin, cyclooxygenase-2, and apoptosis in colorectal cancer. Int J Colorectal Dis.

[36] Breuer-Katschinski B, Nemes K, Marr A, Rump B, Leiendecker B, Breuer N, Goebell H (1999). Helicobacter pylori and the risk of colonic adenomas. Colorectal Adenoma Study Group. Digestion.

[37] Shmuely H, Passaro D, Figer A, Niv Y, Pitlik S, Samra Z, Koren R (2001). Relationship between Helicobacter pylori CagA status and colorectal cancer. Am J Gastroenterol.

